# Effects of bushen qianggu method for primary osteoporosis: A protocol for systematic review and meta-analysis

**DOI:** 10.1097/MD.0000000000020697

**Published:** 2020-06-12

**Authors:** Guocai Chen, Yingxin Guan, Xiangling Ye, Guoqian Chen, Jianping Du, Wengang Liu, Chuanxi Zhao, Nan Yao, Xuemeng Xu

**Affiliations:** a The Fifth Clinical Medical School, Guangzhou University of Chinese Medicine.; b Guangdong Second Traditional Chinese Medicine Hospital; c Guangdong Provincial Key Laboratory of Research and Development in Traditional Chinese Medicine, Guangdong Province Engineering Technology Research Institute of Traditional Chinese Medicine, Guangzhou, Guangdong, China.

**Keywords:** bushen qianggu, meta-analysis, primary osteoporosis, protocol, systematic review

## Abstract

**Background:**

Primary osteoporosis (POP) is one of the most common orthopedic diseases with a high risk of fracture. Effective treatment of POP is of great significance to reduce the rate of disability and improve the quality of life. Bushen qianggu (BSQG) is a classical method of TCM in treating POP. However, there is no systematic review related to BSQG for POP. The purpose of this study is to provide a comprehensive and reliable evaluation of the clinical evidence of BSQG in the treatment of POP.

**Methods and analysis:**

Relevant randomized controlled trial literature evaluating the effect of BSQG on patients with POP will be obtained by searching the PubMed, Embase, MEDLINE, Cochrane Library Central Register of Controlled Trials, China national knowledge infrastructure database, Wan fang database, Chongqing VIP information, and SinoMed from their inception to May 2020. Two researchers will select and evaluate qualified studies independently. The bone mineral density value and the incidence of fractures will be accepted as the primary outcomes. The meta-analyses will be performed by using the RevMan 5.3.

**Results:**

This study will provide a comprehensive evaluation of the efficacy and safety of BSQG method for patients with POP.

**Conclusion:**

The conclusion of our systematic review will provide evidence to judge whether BSQG is an effective intervention for patients with POP.

Trial registration number: 10.17605/OSF.IO/ZMX3W.

## 1 Introduction

Primary osteoporosis (POP) is defined as a pathologic unbalancing of bone resorption and formation that leads to bone fragility and an increased risk of fractures.^[1,2]^ It is a worldwide health problem with serious consequences and a heavy financial burden.^[3]^ Moreover, it is one of the main causes of disability and death in elderly patients, especially with the advent of an aging society.^[4]^ According to the National Health and Nutrition Examination survey in China, the prevalence of osteopenia in people aged 50 years or older is about 64.6% and 57.6% in women and men, respectively.^[5]^ Therefore, early and effective treatment of POP is of great significance to reduce the rate of disability and improve the quality of life. At present, the treatment of POP mainly includes bisphosphonates, estrogen, raloxifene, denosumab, and so forth.^[6,7]^ Other treatments include calcium and vitamin D are also recommended as the basic treatment for POP. These therapies have shown good efficacy in the treatment of patients with POP. However, due to the lifelong treatment requirements, high cost, and the potential negative side effects,^[8–10]^ seeking complementary and alternative treatments for POP is still pertinent.

Traditional Chinese medicine (TCM), a vital part of complementary and alternative medicine, is wildly used in China for its multitarget and fewer side effects. Clinical practice and animal experiments have proved that TCM has a positive therapeutic effect on osteoporosis.^[11]^ In the guidelines for diagnosis and treatment of senile osteoporosis in 2018 in China, Qianggu capsule, a Chinese patent medicine, was recommended as a drug for POP.^[12]^ According to the characteristics of TCM-defined syndromes, Shen deficiency was considered the major pathogenesis of POP.^[13]^ Therefore, the bushen qianggu (BSQG) method should be the principal method in treating POP. Currently, TCMs such as Zhuangguqiangjin tablets, Bushen Zhuanggu granules, and Qianggu capsules, which use the method of BSQG, have been proved to be effective in the therapy of POP.^[14,15]^ However, there is still a lack of high-quality evidence to support the effectiveness and safety of BSQG on patients with POP. In this work, we will perform a systematic review to evaluate the efficacy and safety of BSQG in the treatment of POP to provide a reference for clinical application.

## 2 Materials and methods

We will refer to the preferred reporting items for the systematic review and meta-analysis (PRISMA) to perform this study.^[16]^ The work has been registered at Open Science Framework (OSF, https://osf.io/). The registration DOI of this study is 10.17605/OSF.IO/ZMX3W.

### 2.1 Inclusion criteria

#### 2.1.1 Type of studies

In this work, we will include all randomized controlled trials (RCTs) which explore the specific efficacy and safety of the BSQG in the treatment of POP. Nonrandomized control studies and observational study will be excluded.

#### 2.1.2 Types of patients

All patients with a confirmed diagnosis with POP that including postmenopausal osteoporosis and age-related osteoporosis, will be included. There will be no limitation about age, gender, region, and other factors.

#### 2.1.3 Types of interventions and comparisons

Patients in the treatment group were treated with BSQG alone or in combination with conventional pharmacotherapies. Control interventions will include no treatment, placebo control, and conventional pharmacotherapies. Studies, where the control group is different from the pharmacotherapy in the treatment group, will be excluded. Pharmacotherapies include drugs recommended by the international or domestic authorized clinical guidelines.

#### 2.1.4 Types of outcomes

The primary outcomes of this review will focus on the improvement of bone mineral density value and the incidence of fractures. The secondary outcomes included the visual analog pain score, urinary calcium creatinine ratio, serum calcium, serum phosphorus, bone gla protein, alkaline phosphatase, quality of life, and adverse events in the treatment.

### 2.2 Search strategy

To identify all relevant studies, we will search the following 8 databases from inception to May 2020: PubMed, Embase, MEDLINE, Cochrane Library Central Register of Controlled Trials, China national knowledge infrastructure database, Wan fang database, Chongqing VIP information, and SinoMed. Besides, we will also search Google scholar, Baidu Scholar to find out other related literature. Two authors (Guocai Chen and Yingxin Guan) will search and screen all the citations independently. A search strategy that combines MeSH terms and free words will be adopted. The search strategy was as follows:

1. Search (((((bushen qianggu[MeSH Terms]) AND bushen[Title/Abstract]) AND qianggu[Title/Abstract]) AND zhuanggu[Title/Abstract]) AND bushen formula[MeSH Terms]) AND tonifying kidney[Title/Abstract].
2. Search ((((osteoporosis[MeSH Terms]) AND Osteoporosis, Postmenopausal[MeSH Terms]) AND primary osteoporosis[Title/Abstract]) AND age-related osteoporosis[Title/Abstract]) AND senile osteoporosis[Title/Abstract].
3. Search ((((((((randomized controlled trial[Title/Abstract]) AND RCT[Title/Abstract]) AND controlled clinical trial[Title/Abstract]) AND randomized[Title/Abstract]) AND randomly[Title/Abstract]) AND random[Title/Abstract]) AND controlled[Title/Abstract]) AND control[Title/Abstract]) AND trial[Title/Abstract].
(1) and (2) and (3)

### 2.3 Study selection and data extraction

#### 2.3.1 Selection of studies

**Figure 1:**
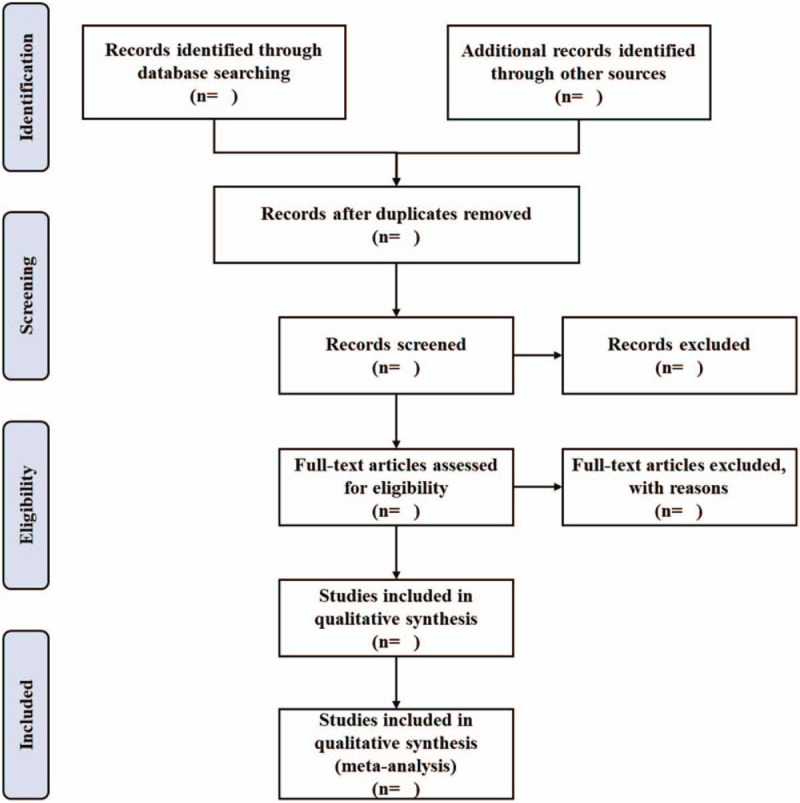
Flow chart of study selection.

We will extract the citations from the above databases by EndNote X9.0 (Stanford, Connecticut, https://endnote.com). Based on the research criteria and search strategies, two reviewers (Guocai Chen and Yingxin Guan) will review the topics and abstracts independently. The eligible articles will be further determined for inclusion by reading the full text. Any different opinions generated between the 2 reviewers will be resolved through discussion with other reviewers. A PRISMA flow chart will be drawn to illustrate the study selection procedure (Fig. 1).

#### 2.3.2 Data extraction and management

We will check the results of the data extraction and provide arbitration for further disagreements. The data of those qualified articles will be export to Microsoft Excel. Then, we will extract and record the first author's name, year of publication, study design, intervention, sample size, duration of intervention, and outcomes. We will contact the corresponding authors for more information if data are missing or unclear.

#### 2.3.3 Assessment of risk of bias

To assess the risk of bias, the Cochrane collaboration's tool will be used. Two reviewers (Guocai Chen and Yingxin Guan) will evaluate the methodologic quality of the RCTs, independently. The risk of bias of a trial will be evaluated through 7 items, including random sequence generation, allocation concealment, blinding of participants and personnel, blinding of outcome assessment, incomplete outcome data, selective reporting, and other sources of bias. The studies will be evaluated as “Low risk,” “High risk,” or “Unclear risk.”

#### 2.3.4 Measures of treatment effect

For dichotomous data, the risk ratio with 95% confidence intervals will be used. For continuous outcomes, the mean difference (MD) or standard MD (SMD) with 95% confidence intervals will be utilized for evaluating the treatment effect.

#### 2.3.5 Assessment of heterogeneity

Heterogeneity will be assessed by Cochrane *X*^2^ and *I*^2^ tests.^[17]^ If *P* ≥ .05 and *I*^2^ ≤ 50%, it suggests that there is no statistical heterogeneity or the heterogeneity is small. If *P* < .05 and *I*^2^ > 50%, it manifests that the study has significant statistical heterogeneity.

#### 2.3.6 Assessment of reporting bias

If there are more than 10 trials included in the study, a funnel plot will be drawn to assess whether there is a publication bias. According to the Egger test, publication bias will be statistical appraised.^[18]^*P* < .05 is considered to have publication bias.

#### 2.3.7 Data synthesis

The RevMan 5.3 software provided by the Cochrane Collaboration will be used for data analysis. If there is no statistical heterogeneity, the fixed effect model will be used. If there is significant heterogeneity between the studies, the random-effects model will be used. Then the subgroup analysis or sensitivity analysis will be performed. If there are enough studies, the metaregression will be conducted to further explore the sources of heterogeneity.

#### 2.3.8 Sensitivity analysis

Sensitivity analysis will be applied to determine the robustness of the results by ruling out studies of low quality and small sample size. In this way, we will be able to assess the impact of low quality and small sample size studies on the overall results.

#### 2.3.9 Grading the quality of evidence

The quality of evidence for all outcomes will be evaluated using the Grading of Recommendations Assessment, Development, and Evaluation (GRADE).^[19]^ In the GRADE system, the quality of evidence will be categorized into 4 levels: high, moderate, low, and very low quality.

#### 2.3.10 Ethics and dissemination

This systematic review will not require ethical approval because there are no data used in our study that are linked to individual patient data. The results will be disseminated only in a peer-reviewed publication.

## 3 Discussion

The POP is one of the most common orthopedic diseases that develop in association with normal processes of menopause and advancing age. It is an important disease affecting the quality of life of patients seriously. Therefore, effective intervention should be conducted in the management of POP. BSQG is a classical method of TCM in treating POP. A series of clinical studies have confirmed the efficacy of BSQG in POP treatment. However, there is no systematic review related to BSQG for POP, which limits the clinical application of this method. In this paper, we present a protocol for a systematic review of the BSQG method for POP. We hope this review will offer reliable references for clinicians in the treatment of POP.

## Author contributions

**Conceptualization:** Wengang Liu, Chuanxi Zhao, Nan Yao, Xuemeng Xu.

**Data curation:** Guocai Chen, Yingxin Guan.

**Formal analysis:** Xiangling Ye, Guoqian Chen.

**Investigation:** Guocai Chen, Yingxin Guan, Xiangling Ye.

**Methodology:** Guocai Chen, Yingxin Guan, Xiangling Ye.

**Software:** Guocai Chen, Yingxin Guan.

**Project administration:** Wengang Liu, Chuanxi Zhao, Nan Yao, Xuemeng Xu.

**Writing – original draft:** Guocai Chen, Yingxin Guan.

**Writing – review & editing:** Jianping Du, Wengang Liu.
